# UNetGE: A U-Net-Based Software at Automatic Grain Extraction for Image Analysis of the Grain Size and Shape Characteristics

**DOI:** 10.3390/s22155565

**Published:** 2022-07-26

**Authors:** Ling Zeng, Tianbin Li, Xiekang Wang, Lei Chen, Peng Zeng, Jason Scott Herrin

**Affiliations:** 1 Geomathematics Key Laboratory of Sichuan Province, Chengdu University of Technology, Chengdu 610059, China; leichen953@gmail.com; 2 State Key Laboratory of Geohazard Prevention and Geoenvironment Protection, Chengdu University of Technology, Chengdu 610059, China; ltb@cdut.edu.cn (T.L.); zengpeng15@cdut.edu.cn (P.Z.); 3 State Key Laboratory of Hydraulics and Mountain River Engineering, Sichuan University, Chengdu 610065, China; wangxiekang@163.com; 4 Facility for Analysis Characterization Testing Simulation, Nanyang Technological University, Singapore 639798, Singapore; jsherrin@ntu.edu.sg

**Keywords:** grain extraction, software, U-Net algorithm, image analysis, grain shape and size

## Abstract

The shape and the size of grains in sediments and soils have a significant influence on their engineering properties. Image analysis of grain shape and size has been increasingly applied in geotechnical engineering to provide a quantitative statistical description for grain morphologies. The statistic robustness and the era of big data in geotechnical engineering require the quick and efficient acquirement of large data sets of grain morphologies. In the past publications, some semi-automation algorithms in extracting grains from images may cost tens of minutes. With the rapid development of deep learning networks applied to earth sciences, we develop UNetGE software that is based on the U-Net architecture—a fully convolutional network—to recognize and segregate grains from the matrix using the electron and optical microphotographs of rock and soil thin sections or the photographs of their hand specimen and outcrops. Resultantly, it shows that UNetGE can extract approximately 300~1300 grains in a few seconds to a few minutes and provide their morphologic parameters, which will ably assist with analyses on the engineering properties of sediments and soils (e.g., permeability, strength, and expansivity) and their hydraulic characteristics.

## 1. Introduction

The size and the shape of natural grains in rocks or sediments usually reflect the mechanical and chemical processes involved in their formation [1]. Grain shape has a significant influence on the mechanical behavior of granular soils; therefore, accurate characterization of sand grain morphology is important in predicting the engineering performance of sand [2,3,4]. Sedimentary grain size is the most important tool in interpreting the nature of source sediments and the dynamic condition of deposition [5,6,7,8]. The grain size distribution of porous sediments and soils is virtually of value in providing initial rough estimates of their engineering properties such as permeability, strength, and expansivity [9,10] in predicting the hydraulic characteristics [11,12,13,14,15,16,17]. Moreover, grain shape and size influence the overall strength of landslide material and its resistance to erosional processes, thus determining the slope stability and governing dynamical properties in surface processes such as avalanches, landslides, and debris flows [17,18,19,20,21].

In recent years, image analysis (IA) of grain size and morphology has been increasingly applied in geotechnical engineering, e.g., [1,22,23,24,25,26,27,28]. IA can generate both grain size and shape distributions and provide a quantitative statistical description for them by digitizing the outlines of grains using photography [29]. Grain size distribution obtained using IA shows a good correlation with available methods such as mechanical sieving and laser diffraction [30,31]. Those methods, including IA, mechanical sieving, and laser diffraction, have their respective advantages and disadvantages: mechanical sieving is most suitable for coarse grains, is lowest cost for analysis, and is the easiest handling instrument, but often makes particles that pass through a sieve actually have one dimension bigger than the size of the sieve apertures; laser diffraction is most suitable for fine grains, is a quick and easy-to-apply technique that determines a size distribution based on the analysis of a huge number of particles, but is expensive for analysis and lays an assumption that all particles are spherical in shape, which, in reality, is almost never the case; IA is suitable for fine-to-coarse grains, is easy to obtain images especially when using optical photography, but is time consuming to obtain statistically significant data and is just directed to obtain a 2D size of particles. Therefore, there are two concerns for IA application to grain size distribution: one is the conversion of three-dimensional (3D) grain size to their 2D sections (or call it “cut effect”: the length of a random thin-section cut is smaller than the real diameter of its 3D grain [32,33]); the other is the development of the automated systems that can analyze a large number of particles quickly and intelligently to obtain statistically significant data.

For the cut effect, two methods have been raised to solve it: (1) firstly, the morphological correction raised by many engineering geologists [28,34,35] was to conduct relations between 3D shapes of some grain samples and their projected 2D sections; (2) secondly, some researchers, e.g., [36,37], preferred Monte Carlo experiments to study grain size distribution using large stochastic simulation datasets, because they thought that such a statistical significance using large stochastic datasets would truly help correction of the grain-size distribution for cut effect. Furthermore, Cheng et al. [33] conducted stochastic simulations and summarized that at least hundreds of grain size datasets would remove the cut effect for the most part, which meant that the correction of the 2D-to-3D conversion could also be more or less solved by requiring the large datasets of grain size. Meanwhile, the era of geoscientific big data requires the larger area data of grain size.

These factors above increasingly require the development of automated systems that have fast and efficient methods for identifying, outlining, and extracting grains from large-area images as the fundamental step in the study of statistically significant grain shape and size distributions. For example, Zeng et al. [31] developed a user-friendly software, CEmin, based on a set of complied MATLAB routines that can simultaneously and quickly extract large numbers of mineral grains from large-area backscattered electron (BSE) imaging. The method of extraction used in CEmin is based on grayscale ranges of mineral grains of interest, using dilation and anti-dilation techniques to distinguish the borderlines between the matrix and the mineral grains. This method is only applicable for grayscale images and typically requires multiple trials and manual interactions to determine the ideal grayscale ranges of grains and the size of dilation, limiting ease, efficiency, and consistency of use.

With the development of deep learning networks, especially convolutional neural networks (CNNs) [38,39], which use convolution and pooling functions to extract new features for analyzing visual imagery, significant differences have manifested in terms of accuracy and effectiveness compared with the conventional networks for image segmentation and rock detection [40]. However, the critical disadvantage for most of CNN learning is that they require a large dataset (often thousands of datasets) for training to produce reliable prediction. The U-Net, based on the fully convolutional network [41,42] and developed for biomedical image segmentation, has its architecture modified and has extended features; resultantly, the U-Net, as a developed CNN, can yield more precise segmentation maps even though working with fewer training data (often a few tens of training data) [43], which are thus more suitable to segment grains from images in this study. Here, we introduce UNetGE, a user-friendly software based on U-Net architecture, complied with Python, and installed on Windows. UNetGE enables non-machine-learning geological experts to detect, outline, and count the shape and size of grains of interest in rocks and soils from large-area images, and to provide the quantitative description of grain morphologic parameters, including area, perimeter, Feret’s diameter, length and width, aspect ratio, and circularity. Compared with CEmin mentioned above [32], UNetGE is not limited to grayscale images and is also ably fit to colored images. UNetGE is not yet limited to plagioclase extraction and even fit to any mineral of igneous, metamorphic, and sedimentary rocks, and UNetGE shows an improvement in extracting a large amount (especially over thousands) of datasets.

In this paper, we use a BSE grayscale image that was used in the publications [32,33], a microphotograph of a sandstone thin section, and a photograph of a sandstone outcrop to test this software’s efficiency.

## 2. Principles of UNetGE

The principles of UNetGE consist of these contents: what kinds of images can be applied to UNetGE and how to obtain them, how to process those images for grain extraction by operating UNetGE, what is the core algorithm of UNetGE and its theory, and how to evaluate results of extraction.

### 2.1. Data Acquisition

The object of this study is to recognize, outline, and extract grains of interest from the matrix through image analysis by deep learning and to provide the quantitative description of morphological parameters of grains by calculating the digitized grain outlines. Microscopy is using a microscope to amplify the view of image grains and matrix of grains that cannot be seen with the naked eye by geological thin sections, including optical, electron, and scanning probe microscopy. Moreover, if the grains in rock outcrops or grains in rock hand specimens are ably seen with the naked eye, it can also allow the common photography (e.g., camera) to image grains and their matrix. Simply speaking, the data of grains and their matrix can be imaged by either microscopic or macroscopic photography. Generally, data used in this study consist of grayscale images and colored images.

### 2.2. Data Processing Workflow

**Figure 1:**
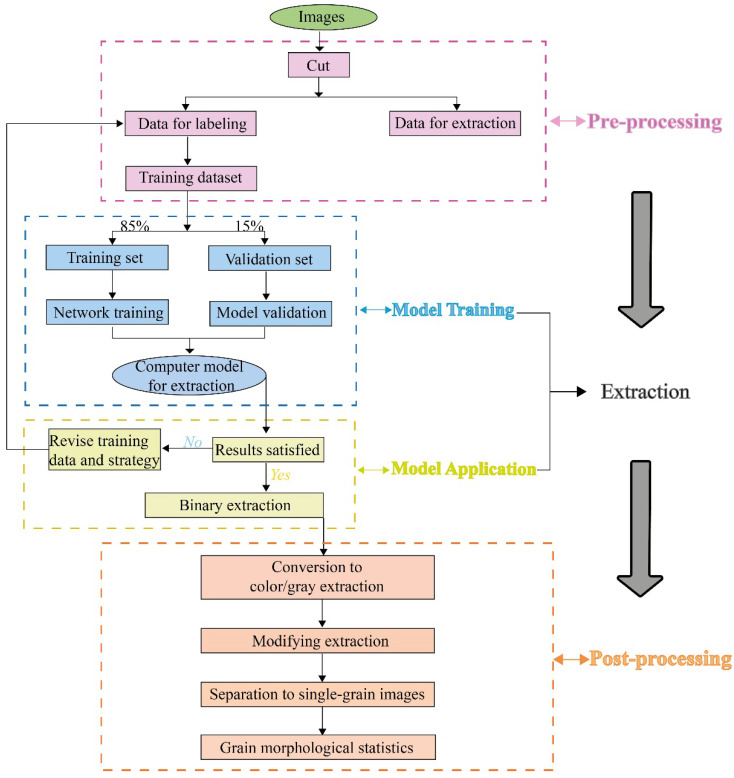
Image processing workflow.

Generally, to digitize and extract outlines of grains and quantitatively describe their morphologies, there are three fundamental steps as shown in Figure 1: (1) The pre-processing step is aimed at preparing dataset for training a net model for extraction (or extraction model). (2) The extraction step is aimed at training the extraction model and applying it to digitize and segment grain outlines from the matrix. (3) The post-processing step is aimed at counting grain morphological parameters based on their digitized outlines.

### 2.3. Methodology of Extraction

The extraction consists of two sub-steps: training an extraction model and then applying this model to extract grains from the matrix by images. The core method is the U-Net algorithm in model training, which applies to general pixel-classification tasks in images with one or multiple channels.

#### 2.3.1. U-Net Architecture

**Figure 2:**
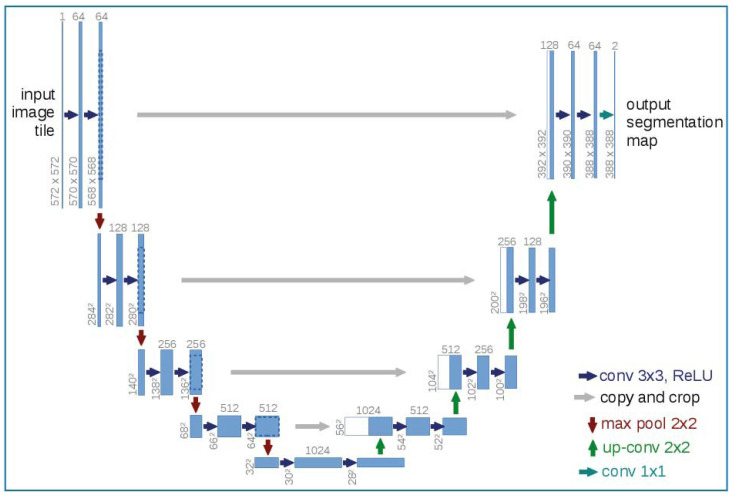
The architecture of the U-Net model and this is reprinted with permission from the reference [43], Copyright 2022, Springer Nature. Each blue box corresponds to a multi-channel feature map. The number of channels is denoted on top of the box. The x-y-size is provided at the lower left edge of the box. White boxes represent copied feature maps. The arrows denote the different operations.

As a kind of fully convolutional network (FCNs), U-Net inherits this characteristic of supplementing a usual contracting network by successive layers, where pooling operators are replaced by upsampling operators to increase the resolution of the output [41,44]. The main modification of U-Net, based on FCNs, is the large number of feature channels in the upsampling part that allows the network to propagate context information to higher resolution layers. Figure 2 shows the architecture of U-Net. A contracting path (left side) and an expansive path (right side) give the u-shaped architecture. The contracting path follows the typical architecture of a convolutional network and the expansive path combines the feature and spatial information through a sequence of upconvolutions and resolution features from the contracting path [43,45].

#### 2.3.2. Model Evaluation Metrics

Furthermore, to evaluate the performance of the extraction model trained by the U-Net algorithm, five metrics are utilized here, including precision, accuracy, recall, F1-score, and loss. Precision is used for finding the correctness of grain recognition; accuracy is the number of correct predictions made as a ratio of all predictions made; recall is used to define how much of the actual grain regions were recognized in the image; F1-score is calculated as the balance between the precision and recall measures; loss is a number indicating how bad the model’s prediction was on a single example. Generally, the better the model’s prediction the smaller the value of loss and the bigger the values of the other four or vice versa.

Loss is the value calculated by the loss function “BCEWithLogitsLoss” here that is within the U-Net architecture and could be referenced in Pytorch [46]. The other four metrics are defined as follows:

(1) $$Precison\;\left(P\right)=\frac{TP}{TP+FP\;}$$

(2) $$Accuracy\;\left(A\right)=\frac{TP+TN}{TP+FP+FN+TN\;}$$

(3) $$Recall\;\left(R\right)=\frac{TP}{TP+FN}$$

(4) $$F1-score=2\times \frac{P\times R}{P+R}$$

where (1) *FP* (false positives) means that pixels belonging to the background were misclassified as belonging to lesions, (2) *FN* (false negatives) means that pixels belonging to lesions were misclassified as belonging to the background, (3) *TP* (true positives) means that pixels belonging to lesions were correctly classified as belonging to lesions, and (4) *TN* (true negatives) means that pixels belonging to the background were correctly classified as belonging to the background [47].

### 2.4. Morphologic Statistics

Generally, the choice of morphologic factors is dependent on the nature of the questions being asked or the field of study in which the grains are being used. This software supplies the calculation and statistics of the following factors:

(1) Area: it is computed and returns the number of non-background pixels in binary image and then can be converted to the real area in unit of micrometer2, or milimeter2, or centimeter2, etc.

(2) Perimeter: it is calculated here as a curve length of the edge of the grain and the number of pixels composing that curve.

(3) Feret’s diameters: they are in fact the caliper diameters [48] and the number of pixels composing those caliper diameters. Thus, the maximum Feret’s diameter (DF_max) is the maximum caliper diameter and the minimum Feret’s diameter (DF_min) is the minimum caliper diameter.

(4) Length and width: length is the maximum distance between any two points on the perimeter of the particle parallel to the major axis and the number of pixels composing that distance; width is the maximum distance between any two points on the perimeter of the particle parallel to the minor axis and the number of pixels composing this distance.

(5) Aspect ratio = minimum Feret’s diameter/maximum Feret’s diameter.

(6) Circularity = $\sqrt{4\pi \cdot \frac{Area}{Perimeter^{2}}}$

Furthermore, the users can also calculate other factors (not listed above) using the digitized outlines of individual grains by our software.

## 3. Overview of Software Layout

**Figure 3:**
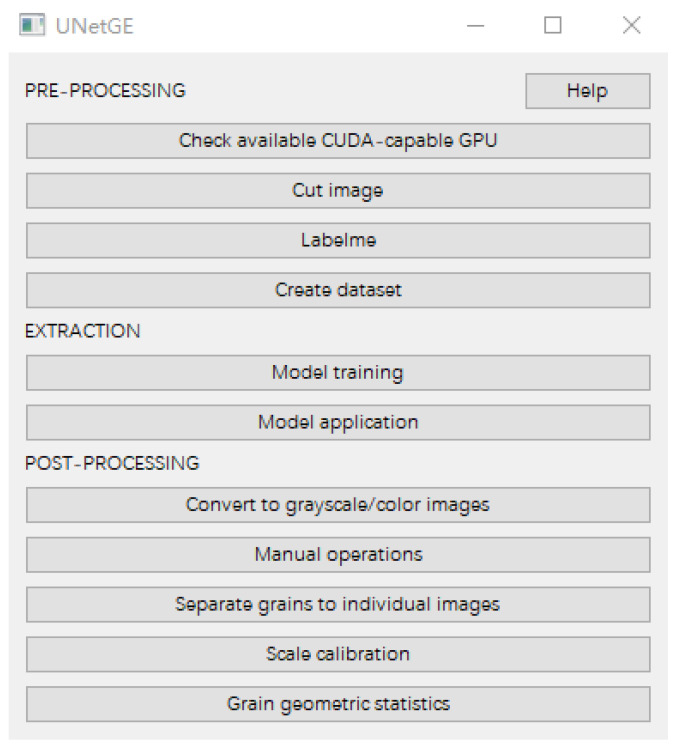
Control panel of UNetGE.

The control panel interface of UNetGE has three subpanels (Figure 3) that correspond with the three fundamental steps in the processing workflow and, thus, eleven utilities to implement all steps and obtain the objects.

(1) “Check available CUDA-capable GPU”: this utility is used to guarantee a running environment of the graphics processing unit (GPU) based on the compute unified device architecture (CUDA), which can help model training much faster than using the central processing unit (CPU).

(2) “Cut image”: this is to cut a large-size image into small-size pieces. For example, a large-area image may be cut into twenty pieces; one piece is used to label grains for training the extraction model, and the remaining pieces are used in model application for extraction.

(3) “Lableme”: this is a project created by the MIT computer science and artificial intelligence laboratory to prepare the training data. The user can use it to label all the grains of interest in one piece of the images and save the result as a JSON (JavaScript object notation) file.

(4) “Create dataset”: this utility works on converting JSON files into a dataset that will be directly used in the extraction model training.

(5) “Model training”: this is utilizing the U-Net algorithm to obtain a fitted model by training a dataset. Parameters of “batch size” and “epoch” need setting to train model. Batch size is at least equal to one and at most equal to the size of the training dataset, while epoch can be set to an integer value between one and infinity. For example, assume a training dataset has 100 samples, a batch size of two, and an epoch of 30, and the model parameters of weights will be updated after each batch of two samples in 50 batches. Therefore, one epoch will involve 50 batches or 50 updates to the model. Therefore, 30 epochs will involve 1500 updates to the model.

(6) “Model application”: this utility is to digitize and extract all grains from the matrix based on the fitted model, and the result is binary extraction.

(7) “Convert to grayscale/color images”: this is to convert those binary extractions to grayscale/color extractions.

(8) “Manual operations”: this consists of two functions of “repair” and “screen”. The “repair” is aimed at refining the digitized outlines of grains. By creating lines with width, some connected grains are separated using “erase” of “repair”; by creating polygons, some unwanted parts of grains may be removed using “erase” of “repair”, and some holes inside grains can be filled using “binary fill” or “gray/color fill” of “repair”. The “screen” is used to screen out grains by size; grains of a size smaller or equal to the selected grain will be eliminated out.

(9) “Separate grains to individual images”: this is used to segment the digitized grains one by one from their extraction images into individual images.

(10) “Scale calibration”: this is used to obtain the ratio between real distance and pixel distance.

(11) “Grain morphologic statistics” is used to count all morphologic parameters of each grain, as mentioned in Section 2.4.

## 4. Applications

**Figure 4:**
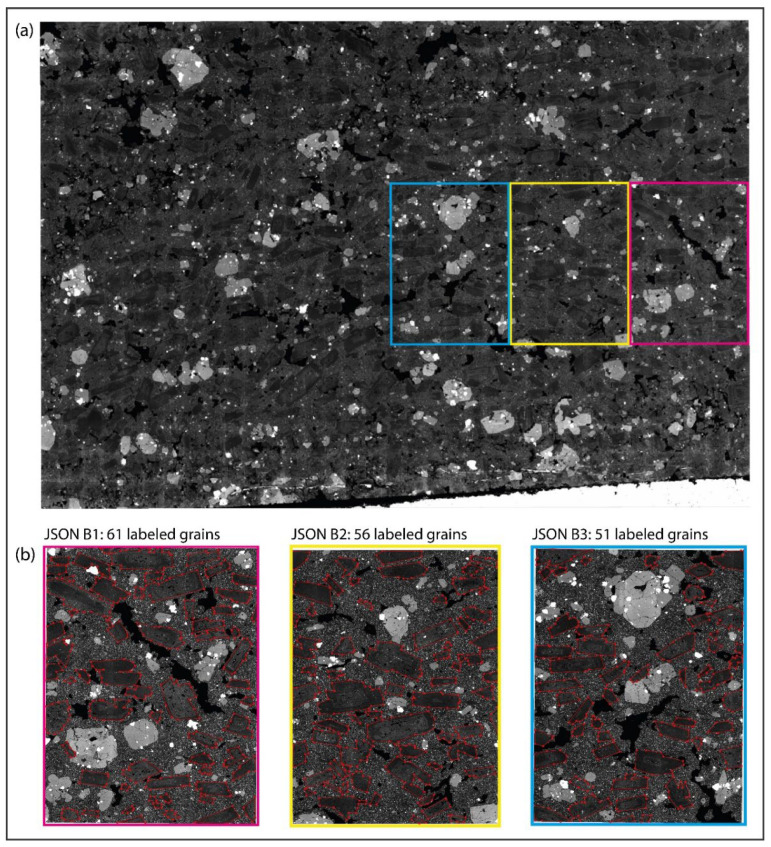
(**a**) The BSE microphotograph of rock thin section. (**b**) The three JSON files where we label mineral grains as the training and validation datasets in model training. The JSON file with the pink edge, yellow edge, and sky blue edge corresponding to the place circled by the pink, yellow, and sky blue rectangles in (**a**).

**Figure 5:**
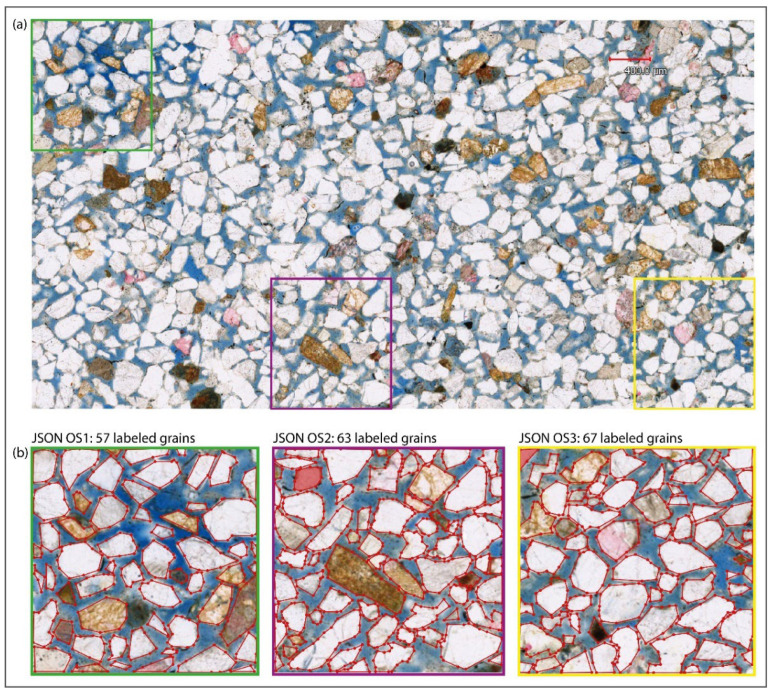
(**a**) The transmitted light microphotograph of offshore sandstone thin section. (**b**) Three JSON files with 57, 63, and 67 labeled grains. The JSON file with the green edge, purple edge, and yellow edge corresponding to the place circled by the green, purple, and yellow rectangles in (**a**).

**Figure 6:**
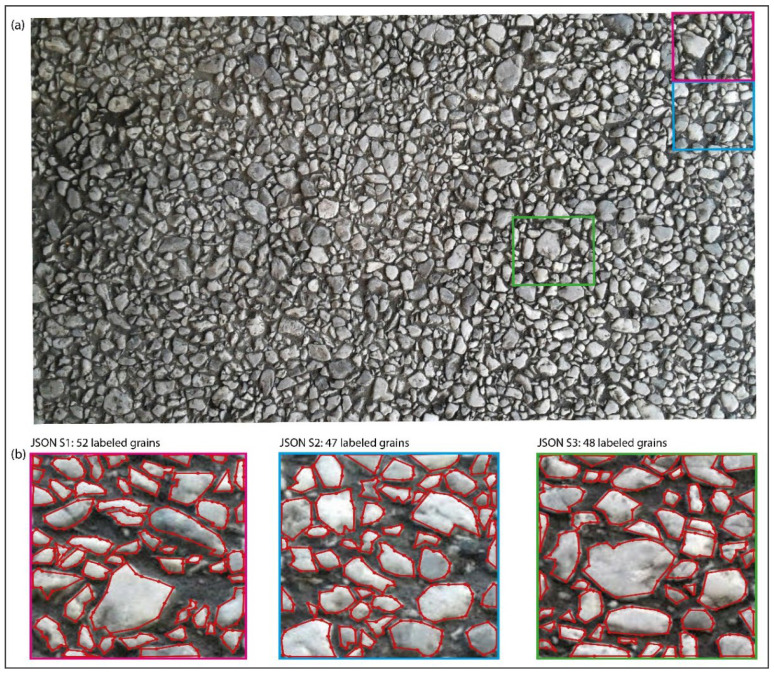
(**a**) The photograph of sandstone outcrop. (**b**) Three JSON files with 52, 47, and 48 labeled gravel grains. The JSON file with the pink edge, sky blue edge, and green edge corresponding to the place circled by the pink, sky blue, and green rectangles in (**a**).

An eight-core 32 GB GPU is used during all processing. Three images are used here as cases: (1) a backscattered electron (BSE) micrograph of rock thin section (Figure 4a) [32,33] with over 600 plagioclase mineral grains; (2) a transmitted-light microphotograph (Figure 5a) of an offshore Miocene sandstone thin section, with about 300 sand grains; (3) a photograph of sandstone outcrop (Figure 6a) with over 1000 coarse grains. Here, we abbreviate the three images respectively as BSE, OFSD, and SAND.

### 4.1. Pre-Processing

The pre-processing is aimed at obtaining training datasets: how to label grains on images and save the results as JSON files, and how to convert the JSON files into training datasets.

#### 4.1.1. Preparing JSON Files

During pre-processing, we make equal-sized cuts for the three images, respectively, and select a few pieces to label the shapes of grains (Figure 4b, Figure 5b and Figure 6b) in Labelme to prepare for the training model in extraction. As a result, there are three JSON files, respectively, for BSE, OFSD, and SAND.

#### 4.1.2. Preparing Datasets

Furthermore, the preparation of training datasets is based on all JSON files (Figure 4b, Figure 5b and Figure 6b). In the applications, we combine JSON files to prepare three groups of datasets with different numbers of training samples and different sizes of training images, respectively, for BSE, OFSD, and SAND, which will be trained as different net models. As a result, Table 1 shows nine groups of datasets and their own JSON files, size of training images, and number of training samples.

### 4.2. Performance of Net Models

In the training, the optimizer used is Pytorch Adam and the default learning rate is 0.01. As mentioned in Section 3, there are two parameters needed for setting the model training, epoch and batch size. Batch size requires huge GPU capability for increasement, especially with the increasement of epoch, and is not easily sensitive to the changes of model performance. For example, the capability of GPU in our application at most allows a batch size of two when epoch is equal to or smaller than 30, and even a batch size of one is just allowed when epoch reaches or over 35; meanwhile, the change of batch size from one to two seems not obviously influencing on five performance metrics (Section 2.3.2) and training time. Therefore, through the entire processing in our applications, batch size is one. In this section, we explore how epoch, number of training samples, and size of training images affect the five metrics of net model performance and the running time to train model.

#### 4.2.1. Effect of Epoch on Model

As shown in Table 1, BSE1, BSE2, and BSE3 have indicated the differences of sample numbers and image sizes for the BSE group; OFSD1, OFSD2, and OFSD3 have indicated those for the OFSD group; SAND1, SAND2, and SAND3 have indicated those for the SAND group. Here, we change epoch values from 5 to 10 to 15 to 20 to 25 to 30 and finally to 35 for the nine datasets (Table 1); thus, there are seven trainings for each dataset.

**Figure 7:**
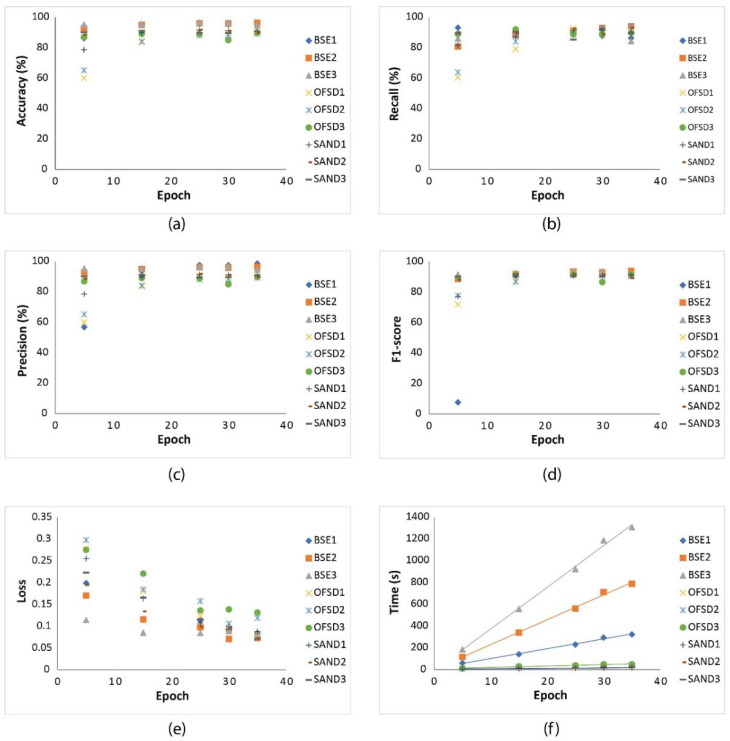
(**a**) The changes of accuracy as the epoch varies from 5 to 35 for the nine groups of BSE1, BSE2, BSE3, OFSD1, OFSD2, OFSD3, SAND1, SAND2, and SAND3. (**b**) The change of recall as the epoch varies from 5 to 35 for the nine groups. (**c**) The change of precision as the epoch varies from 5 to 35 for the nine groups. (**d**) The changes of F1-score as the epoch varies from 5 to 35 for the nine groups. (**e**) The changes of loss as the epoch varies from 5 to 35 for the six groups. (**f**) Time of training changes vs. epoch changes for the nine groups.

Figure 7 shows how epoch correlates with five performance metrics and training time under different sample numbers and image sizes for BSE, OFSD, and SAND. Generally, Figure 7a–d show that accuracy, precision, recall, and F1-score become relatively stable after an epoch of 20; Figure 7e shows loss is stable after an epoch of 30; Figure 7f shows that time of training is linearly correlated with epoch.

#### 4.2.2. Effects of Sample Numbers and Image Sizes on Model

Based on the results in Section 4.2.1, considering time cost and model robustness, we assume a constant epoch of 30 to train the nine datasets to see how the different numbers of training samples and the different sizes of training images affect the performance of net models and the training time. Table 2 show the resultant performance of the nine net models.

**Figure 8:**
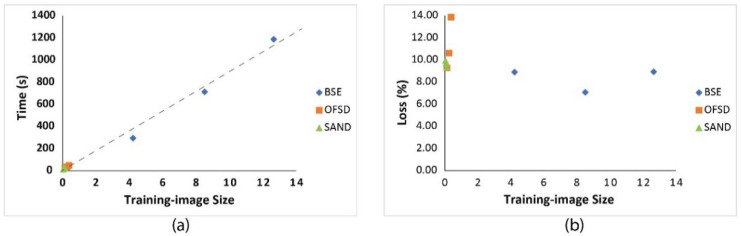
(**a**) Time changes vs. size changes of training images for the nine groups. (**b**) Loss changes vs. size changes of training images for the nine groups.

Based on the analysis of results in Table 2, it seems that the number of training samples is uncorrelated with training time, but the training-image size is obviously correlated with training time as shown in Figure 8a. Moreover, it is hard to correlate the precision, accuracy, recall, and F1-score to either the number of training samples or the size of training images, but the loss seems related to the magnitude of training-image size: e.g., the loss related to the size below 1 MB is generally bigger than that related to the size above 1 MB as shown in Figure 8b.

#### 4.2.3. Summary and Discussion

According to the results shown in Table 2, the five metrics of performance of the nine trained models based on the nine training datasets are generally acceptable for application, because all accuracy, precision, recall, and F1-score are above 80% and all loss are below 14%. Moreover, models with training sample numbers of 50~60 have the similar performance with or even better than models with sample numbers of over 100. Therefore, considering the cost of time and labor to label the grains, we recommend the number of training samples of 50~100.

Moreover, the models’ performance in the OFSD and SAND groups are generally lower than those in the BSE groups. Indeed, based on comparison among Figure 4, Figure 5 and Figure 6, it is obvious that the resolution and quality of OFSD photographs (Figure 5) and SAND photographs (Figure 6) is lower than that of BSE images (Figure 4), and the grains in Figure 4 are clearer and more obvious in geometric features than those in Figure 5 and Figure 6. Generally, the first-rate factor in determining the model performance is the resolution and quality of images used in the training and the distinguishing geometric features of grains in the images. However, the higher the resolution and the quality of images, the bigger the sizes of those images. Furthermore, although the five metrics of the model performance of all BSE groups is generally a little better than those of the OFSD groups and SAND groups, the training time of BSE groups is much more than those of the OFSD and SAND groups. Summarily, once the geometric features of grains are clear for recognition, it is not necessary to use images of much higher resolution and quality as this would lead to their increasing size and thus increasing training time.

### 4.3. Results of Extraction and Statistics

**Figure 9:**
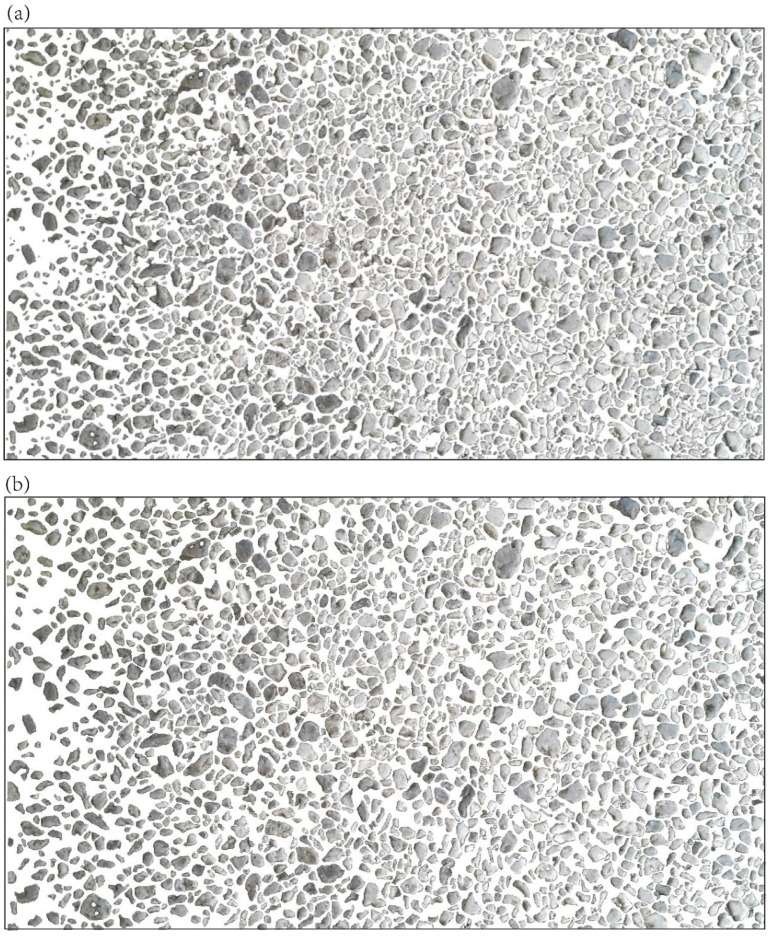
(**a**) The resultant color image converted from its binary-extraction image by the photograph of a sandstone outcrop in the model application. (**b**) The modified image is based on (**a**) using the utility of manual operation.

From a comprehensive consideration of training time, model performance, and the labor and time to label the outlines of grains, model BSE1 is optimal for grain extraction by BSE image, model OFSD1 is optimal for grain extraction by OFSD image, and model SAND1 is optimal for grain extraction by SAND image. Here, we take the SAND1 extraction result as an example that is shown in Figure 9.

**Figure 10:**
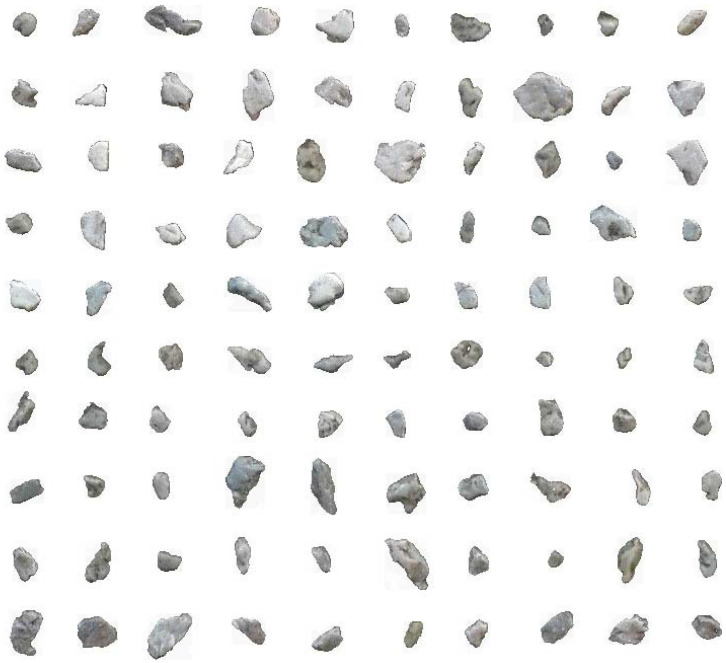
A total of 100 examples of a total of 1311 single-grain images segmented from the modified image of Figure 9b.

Figure 9a is the gray conversion of binary extraction from the step of model application. Figure 9b is the modification based on Figure 9a by manual operations. Figure 10 shows 100 examples of 1311 single-grain images that are separated from Figure 9b. Table 3 shows the resultant examples of counting grain morphologic parameters by the utility of grain morphologic statistics.

### 4.4. Comparisons with CEmin

**Figure 11:**
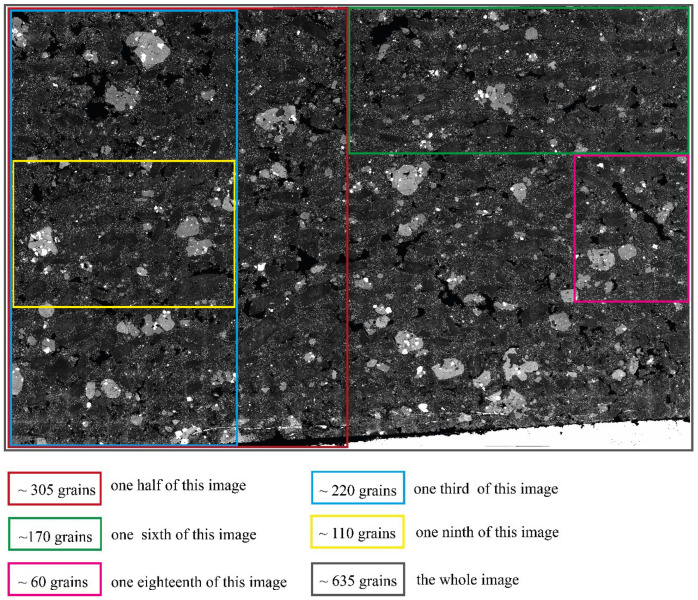
Five kinds of equidimensional cuts of the BSE microphotograph of rock thin section with different numbers of plagioclase mineral grains.

CEmin is just for the processing of grayscale images, whereas UNetGE can process not only the grayscale ones but also the colored ones. Therefore, we take the BSE group that is grayscale (Figure 4) for the comparison of performances between CEmin and UNetGE. We equidimensionally cut the BSE image of plagioclase by five ways; thus, there are five different sizes as shown in Figure 11. The pink rectangle piece in Figure 11 is the same as JSON B1 in Figure 4 that is one-eighteenth part of this image; the yellow rectangle piece is one-ninth part of this image; the green rectangle piece is one-sixth part of this image; the blue rectangle piece is one-third part of this image; the red rectangle piece is one-second part of this image; the whole image is rectangle in grey.

CEmin is utilized to extract the six pieces of image, respectively. UNetGE utilizes the pink rectangle piece for model training as before, then the trained model is applied to extract the other five pieces, respectively. Generally, the pre-processing of CEmin includes removing voids and repeating trials to set parameters. The time to repeat trials for setting parameters is hard to estimate and it depends on user experience, but generally it ranges from tens of seconds to a few minutes. The time to remove voids and the time to extract grains by CEmin is listed in Table 4. The pre-processing of UNetGE includes cutting images, confirming the running environment, and labeling grains to prepare for training datasets. The time for cutting images and confirming the running environment takes rather less time (a few seconds) and can be ignored, and the time to label grains would take about 10 min to label 61 grains (JSON B1 in Figure 4) and about a few seconds to create them to dataset. The time to train a model (model BSE1 as listed in Table 2) using the dataset of JSON B1 takes about 294 secs or 4.9 min (Table 2), and the time to extract the other five pieces of image using the trained BSE1 model is listed in Table 4.

**Figure 12:**
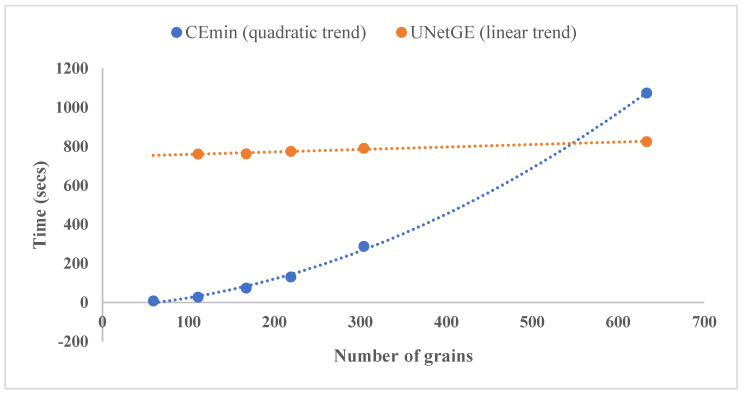
A comparison of time to extract grains between CEmin and UNetGE.

From Table 4, as well as Table 2, it shows that the time to extract grains by CEmin is mainly determined directly by time for extraction, whereas the time to do that by UNetGE is mainly determined by both labeling grains and training models, and the time for model application (a few seconds to over one minute) almost contributes little. Figure 12 is a plot of the numbers of extracted grains vs. the time to extract grains, which shows a quadratic increasement of time with the increasing number of grains using CEmin, and a slow-slope but large-intercept (including 10 min to label grains and 4.9 min to train models) linear increasement of time with the increasing number of grains using UNetGE. Furthermore, Figure 12 shows that with the number of grains over 550, the time by UNetGE to extract grains becomes increasingly advantageous compared with CEmin, and CEmin prefers to extract grains with an amount of less than 500.

**Figure 13:**
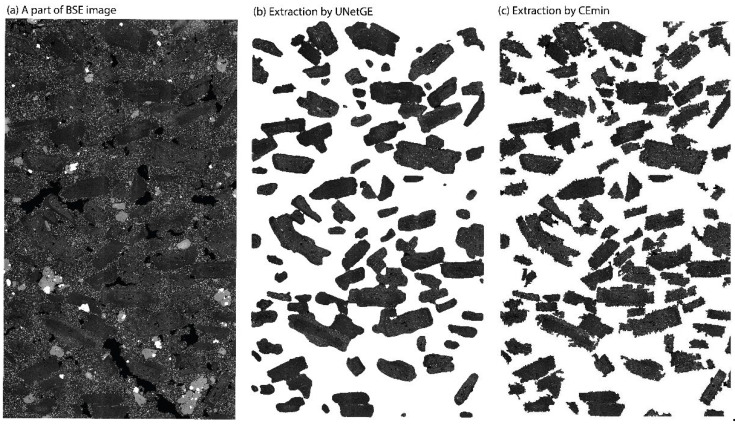
(**a**) A part of BSE image. (**b**) The direct grayscale extraction of this part of BSE without manual correction by UNetGE based on the trained model of BSE1 (Table 2). (**c**) The direct grayscale extraction of this part of BSE without manual correction by CEmin.

Figure 13 shows a contrast of the direct grayscale extraction of the BSE microphotograph between CEmin and UNetGE and a contrast of the two extractions with the original BSE part. From the perspective of grain shape, the grains extracted by UNetGE have smoother outlines than those extracted by CEmin. Furthermore, the performance metrics of extraction by CEmin in term of “precision”, “recall”, and “F1-score” are, respectively, 93.67%, 96%, and 94.82%, which are compared with extraction by UNetGE based on the BSE1 model (97.36%, 87.84%, and 92.36% as shown in Table 2). Generally, although the performance metrics by CEmin are a bit better than those by UNetGE, we still consider that such a difference of metrics cannot produce substantial influences on the extraction performances. However, the more excellent the shape of grains extracted by UNetGE is the more important for the output, because it would save the time of post-processing (e.g., the manual modification of grain shape).

As for these steps of “POST-PROCESSING”, the time for “convert to grayscale/color images”, “separate grains to individual images”, “scale calibration”, and “grain morphologic statistic” is in the magnitude of seconds and can thus be neglected. In “manual operations”, the time for screening out grains with an area below an assumed threshold by “screen” has a few seconds, but the time for separating the connected grains and filling the holes inside grains by “repair” has a few minutes to tens of minutes, which depends on the shape of grains extracted. Furthermore, compared with CEmin, the “manual operations” by UNetGE just adds a function of “screen”, and the time to separate connected grains and fill holes by UNetGE is similar to that by CEmin.

## 5. Conclusions

Generally, UNetGE can use small-size training samples (e.g., tens of grain samples), compared with most CNNs, to reach a clear recognition and quick extraction of grain outlines, provided that the size of training images is not too big, the quality of the image is good, and the features of the grain shapes are remarkable.

Compared with the previous version of CEmin [32]: firstly, UNetGE shows an extreme improvement in large-data extraction (Figure 12), especially when the number of grains is over a few thousand, UNetGE could still carry out a fast extraction using a model trained just by tens of grains; secondly, CEmin is nowadays used just for plagioclase mineral extraction by grayscale images, whereas UNetGE can extract any kind of mineral grain by grayscale images; thirdly, besides grayscale microphotographs, UNetGE can also recognize and extract grains by optical microphotographs, hand specimens, and outcrop photographs that are all colored; fourthly, UNetGE may identify grains with more complicated features (e.g., parts of grains in offshore sandstone microphotographs are oxidized and thus turn a bit yellow as shown in Figure 5).

## 6. Patents

The software patent No. is 202210343471.7 issued by China National Intellectual Property Administration.

## Figures and Tables

**Table 1:** The descriptions of datasets for models training.

Dataset ID	JSON Files Included	Total Size of Training Images Included (MB)	Total Number of Training Samples Included
BSE1	JSON B1	4.22	61
BSE2	JSON B1, JSON B2	8.52	117
BSE3	JSON B1, JSON B2, JSON B3	12.66	168
OFSD1	JSON OS1	0.127	57
OFSD2	JSON OS1, JSON OS2	0.255	120
OFSD3	JSON OS1, JSON OS2, JSON OS3	0.380	187
SAND1	JSON S1	0.038	52
SAND2	JSON S1, JSON S2	0.075	99
SAND3	JSON S1, JSON S2, JSON S3	0.112	147

**Table 2:** Resultant time and performance for nine net models under an epoch of 30.

Model ID	Sample Number	Size (MB)	Time (s)	Loss (%)	Accuracy (%)	Precision (%)	Recall (%)	F1-Score (%)
BSE1	61	4.22	294	8.89	95.28	97.36	87.84	92.36
BSE2	117	8.52	712	7.08	95.86	92.93	92.93	92.93
BSE3	168	12.66	1186	8.93	96.52	96.08	91.02	93.48
OFSD1	57	0.127	25	9.28	89.89	91.38	91.15	91.27
OFSD2	120	0.255	34	10.62	88.27	88.78	92.73	90.71
OFSD3	187	0.38	48	13.87	84.93	84.26	89.08	86.6
SAND1	52	0.038	13	9.94	91.08	90.34	91.49	90.91
SAND2	99	0.075	25	9.71	91.06	94.72	88.71	91.62
SAND3	147	0.112	23	9.32	89.74	88.66	92.34	90.46

**Table 3:** The statistical results of morphologic parameters of 20 grains (the first 10 grains and the last 10 grains shown in Figure 10).

Filename	DF_min (Pixel)	DF_max (Pixel)	Width (Pixel)	Length (Pixel)	Perimeter (Pixel)	Area (Pixel)	DF_min (mm)	DF_max (mm)	Width (mm)	Length (mm)	Perimeter (mm)	Area (mm)	Aspect ratio	Circularity
sands_6	21	23	25	26	75	379	0.424	0.453	0.500	0.520	1.500	0.151	0.938	0.919
sands_15	19	30	22	34	86	401	0.379	0.609	0.440	0.680	1.729	0.160	0.622	0.821
sands_17	23	55	27	58	151	866	0.462	1.098	0.540	1.160	3.024	0.346	0.421	0.690
sands_19	27	28	31	32	96	586	0.540	0.560	0.620	0.640	1.917	0.234	0.964	0.895
sands_28	28	39	31	41	119	733	0.565	0.776	0.620	0.820	2.378	0.293	0.728	0.807
sands_42	26	41	28	43	115	749	0.518	0.819	0.560	0.860	2.303	0.299	0.632	0.842
sands_71	16	34	18	36	86	421	0.311	0.679	0.360	0.720	1.715	0.168	0.458	0.848
sands_80	22	27	25	33	91	438	0.445	0.538	0.500	0.660	1.830	0.175	0.828	0.810
sands_98	32	35	32	45	119	779	0.639	0.690	0.640	0.900	2.371	0.312	0.926	0.834
sands_1139	20	23	24	28	76	324	0.394	0.465	0.480	0.560	1.514	0.129	0.846	0.842
sands_1180	22	35	25	37	98	510	0.446	0.698	0.500	0.740	1.962	0.204	0.639	0.816
sands_1182	20	30	22	33	86	393	0.392	0.604	0.440	0.660	1.717	0.157	0.649	0.818
sands_1191	21	27	22	30	81	380	0.410	0.537	0.440	0.600	1.625	0.152	0.763	0.850
sands_1201	36	37	39	39	133	922	0.720	0.740	0.780	0.780	2.661	0.369	0.973	0.809
sands_1205	20	35	23	37	97	545	0.400	0.695	0.460	0.740	1.943	0.218	0.576	0.851
sands_1214	20	48	22	50	123	581	0.394	0.957	0.440	1.000	2.466	0.232	0.411	0.693
sands_1226	25	33	25	38	106	527	0.495	0.656	0.500	0.760	2.113	0.211	0.755	0.770
sands_1284	21	26	21	31	80	389	0.411	0.512	0.420	0.620	1.609	0.156	0.802	0.869
sands_1292	23	28	27	30	86	491	0.467	0.552	0.540	0.600	1.722	0.196	0.846	0.912

Note: The scaling ratio between pixels and real distance used here is 1 mm equal to 50 pixels length.

**Table 4:** The time to extract grains using CEmin and UNetGE, respectively.

	CEmin				UNetGE
Sub-Group	Setting Number of Voids	Time to Remove Voids (Secs)	Number of Grains Directly Extracted	Time for Extraction (Secs)	Time for Model Application (Secs)
one-eighteenth part	40	4	59	9	It is used to train model about 294 secs
one-ninth part	80	4	111	28	11
one-sixth part	120	6	167	75	13
one-third part	160	11	219	132	25
one-second part	200	19	304	288	41
the whole image	250	43	633	1074	75

## Data Availability

The software and its user’s manual are deposited in GitHub and they can be downloaded by https://github.com/linee1203/UnetGE (accessed on 16 May 2022).
